# Combining targeted panel-based resequencing and copy-number variation analysis for the diagnosis of inherited syndromic retinopathies and associated ciliopathies

**DOI:** 10.1038/s41598-018-23520-1

**Published:** 2018-03-27

**Authors:** Iker Sanchez-Navarro, Luciana R. J. da Silva, Fiona Blanco-Kelly, Olga Zurita, Noelia Sanchez-Bolivar, Cristina Villaverde, Maria Isabel Lopez-Molina, Blanca Garcia-Sandoval, Saoud Tahsin-Swafiri, Pablo Minguez, Rosa Riveiro-Alvarez, Isabel Lorda, Rocío Sanchez-Alcudia, Raquel Perez-Carro, Diana Valverde, Yichuan Liu, Lifeng Tian, Hakon Hakonarson, Almudena Avila-Fernandez, Marta Corton, Carmen Ayuso

**Affiliations:** 1 Department of Genetics, Instituto de Investigaciones Sanitarias – Fundacion Jiménez Díaz University Hospital (IIS-FJD-UAM), Madrid, Spain; 2 Centre for Biomedical Network Research on Rare Diseases (CIBERER), ISCIII, Madrid, Spain; 3 Universidade de Mogi das Cruzes, São Paulo, Brazil; 4 Department of Ophthalmology, Fundacion Jiménez Díaz University Hospital, Madrid, Spain; 5 Department of Biochemistry, Genetics and Immunology, Faculty of Biology, Universidad de Vigo, Vigo, Spain; 6 Center for Applied Genomics, Abramson Pediatric Research Center, The Children’s Hospital of Philadelphia, Philadelphia, PA USA; 7 Medical Scientist Training Program, Perelman School of Medicine, University of Pennsylvania, Philadelphia, PA USA; 8 Division of Human Genetics, Children’s Hospital of Philadelphia, Philadelphia, PA USA; 9 The Perelman School of Medicine, University of Pennsylvania, Philadelphia, PA USA

## Abstract

Inherited syndromic retinopathies are a highly heterogeneous group of diseases that involve retinal anomalies and systemic manifestations. They include retinal ciliopathies, other well-defined clinical syndromes presenting with retinal alterations and cases of non-specific multisystemic diseases. The heterogeneity of these conditions makes molecular and clinical characterization of patients challenging in daily clinical practice. We explored the capacity of targeted resequencing and copy-number variation analysis to improve diagnosis of a heterogeneous cohort of 47 patients mainly comprising atypical cases that did not clearly fit a specific clinical diagnosis. Thirty-three likely pathogenic variants were identified in 18 genes (*ABCC6, ALMS1, BBS1, BBS2, BBS12, CEP41, CEP290, IFT172, IFT27, MKKS, MYO7A, OTX2, PDZD7, PEX1, RPGRIP1, USH2A, VPS13B, and WDPCP*). Molecular findings and additional clinical reassessments made it possible to accurately characterize 14 probands (30% of the total). Notably, clinical refinement of complex phenotypes was achieved in 4 cases, including 2 *de novo OTX2*-related syndromes, a novel phenotypic association for the ciliary *CEP41* gene, and the co-existence of biallelic *USH2A* variants and a Koolen-de-Vries syndrome–related 17q21.31 microdeletion. We demonstrate that combining next-generation sequencing and CNV analysis is a comprehensive and useful approach to unravel the extensive phenotypic and genotypic complexity of inherited syndromic retinopathies.

## Introduction

Inherited syndromic retinopathies (ISR) are clinically and genetically heterogeneous diseases in which retinal alteration is accompanied by systemic anomalies that can involve one or more systems, such as auditory, neurological, musculoskeletal, renal, cardiac, and hepatic systems^1,2^. These disorders can be inherited in an autosomal recessive, autosomal dominant, X-linked, or mitochondrial fashion. Triallelism and oligogenic forms have also been described^3^. To date, ISRs have been related to at least 90 genes based on the Retinal Information Network database (RetNet, https://sph.uth.edu/retnet/).

ISRs include clinically well-defined syndromes such as ciliopathies^4^ (Alström^5^, Bardet-Biedl^6^, Joubert^7^, Senior-Løcken^8^, and Usher syndrome^9^), syndromes related to other cellular components (Cohen syndrome^10^ and peroxisome biogenesis disorders [PBDs]^11^), and even extracellular molecular defects (Stickler syndrome)^12^. In other cases, patients present with diverse non-specific systemic symptoms and signs in which a clear clinical diagnosis cannot be easily achieved^13^. As many of these entities share medical and molecular features that hinder their clinical management and follow-up^13^, ISR diagnosis is quite difficult and challenging, and patients often visit various specialists over a period of years without a conclusive diagnosis being made. Thus, molecular characterization is essential for an accurate and definitive diagnosis and helps to guide reproductive risk assessment, counseling, surveillance, management, and prognosis^1,13^. Our study may also provide clues for appropriate selection of patients to be enrolled in future clinical trials such as those examining gene-based therapies.

Traditional methods for mutation screening are costly and labor-intensive and entail a low molecular diagnostic rate, even for patients with a clear suspicion of ciliopathy^14,15^. Next-generation sequencing (NGS) is a cost-effective approach for the genetic diagnosis of retinal diseases^16,17^. Several studies have focused on specific retinal ciliopathies, such as Bardet-Biedl syndrome (BBS)^18^ and Joubert syndrome^7,19–21^, with a wide range of reported mutation detection rates depending on the number of targeted genes, NGS platform, and use of pre-screened or “naive” cases. In patients with complex atypical phenotypes, chromosomal microarray analysis may also be very useful for assessing contiguous gene deletion syndromes as a cause of disease co-occurrence^22^. To date, few authors have studied the role of copy number variations (CNVs) in retinal dystrophies (RD)^23–27^. We performed a molecular analysis using a custom targeted resequencing approach combined with CNV analysis in order to characterize a heterogeneous cohort of ISR patients whose specific disease had not yet been determined. Our results provide new insights into the genetic complexity of these diseases.

## Results

A cohort of 47 genetically uncharacterized ISR cases underwent a comprehensive analysis of single-nucleotide variants (SNVs) and CNVs, based mainly on a custom targeted NGS for 121- gene panel of ISR-associated and candidate genes (Supplementary Table S1). The genes were selected based on previous associations with ISR reported in the literature, many of which showed a high proportion of associated phenotypes and appeared to be functionally related (Supplementary Fig. S1).

**Figure 1:**
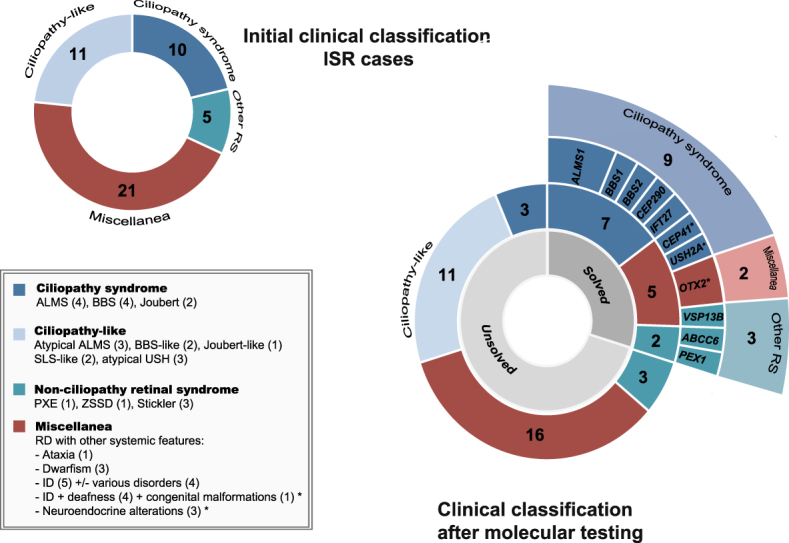
Clinical classification of patients with inherited syndromic retinopathies (ISR) and molecular findings obtained in this study. The two charts summarize initial and reassessed clinical classifications, before and after molecular testing, respectively. Four main phenotypic categories of cases were considered: i) clinically-defined ciliopathy syndromes, represented in dark blue color; ii) ciliopathy-like cases, in which retinal degeneration was found in association with multiple ciliopathy-like features (in light blue color); iii) patients with a clear diagnosis of a non-ciliopathy RD, represented in green, and iv) miscellaneous cases (in red color) with a variety of phenotypes that involved RD with one or more unspecific systemic symptoms, in which an obvious diagnosis could not be clearly established. The different levels of circles of the right chart (from inner to outside) reflect molecular yield depending on these 4 clinical categories and the mutated genes. Inner circle in grey shapes represents the fraction of solved (30%, 14/47) *vs* unsolved (70%, 33/47) cases after molecular testing. Second circle indicates the yield of molecular characterization based on the initial classification. Third level shows the causal genes found in this study and their respective color indicate the associated syndromic retinal disease. Outer level indicated the final classification of the solved cases. *CEP41, *USH2A*, and *VSP13B* cases, which were initially included in the miscellanea group, were re-classified after genetic testing. Dual diagnosis of *USH2A* biallelic pathogenic variants and *de novo* 17q21.31 mosonomy associated with Usher and Koolen de Vries syndromes, respectively. ALMS: Alström syndrome; BBS: Bardet-Biedl Syndrome; ID: Intellectual Disability; ISR: inherited syndromic retinopathies; RD: Retinal Dystrophy; SLS: Senior-Løcken syndrome; USH: Usher syndrome; ZSSD: Zellweger syndrome spectrum disorder.

Patients were classified into 4 major phenotypic categories based on clinical features and suspected clinical diagnosis (Fig. 1): i) ciliopathies, including 10 cases with Alström, BBS, or Joubert syndrome; ii) ciliopathy-like, including 11 patients with RD and some ciliopathy-related systemic anomalies (polydactyly, obesity, diabetes, hearing, loss, and/or nephronophthisis); iii) other known syndromic retinopathies, a total of 5 patients with a clear diagnosis of either PBDs, Stickler syndrome, or pseudoxanthoma elasticum (PXE); iv) miscellanea, including 21 cases with heterogeneous phenotypes involving retinal disease with 1 or more non-specific systemic features, such as congenital malformations, ataxia, dwarfism, intellectual disability (ID), or neuroendocrine alterations.

A total of 33 pathogenic or likely pathogenic variants were identified in 49% of patients (23/47) (Tables 1 and 2 and Supplementary Table S2), including 30 SNVs and 3 CNVs in 18 different genes (*ABCC6, ALMS1, BBS1, BBS2, BBS12, CEP41, CEP290, IFT172, IFT27, MKKS, MYO7A, OTX2, PDZD7, PEX1, RPGRIP1, USH2A, VPS13B, and WDPCP*). Thirteen of the SNVs were novel (Supplementary Table S3), including 2 non-synonymous and 2 in-frame variants (*ABCC6*: p.(Leu495del), *BBS12*: p.(Ser440del), *CEP41:* p.(Ser2Phe), *IFT27:* p.(Tyr35Cys)), which were considered potentially pathogenic based on *in silico* prediction, evolutionary conservation of the wild-type residues, previous knowledge of ISR-associated genes, and disease co-segregation.

**Table 1:** Clinical and genetic information of cases carrying likely disease-causal variants.

Family	Initial Dx	Final Dx	Clinical Features	*Gene*	Allele 1	Allele 2	Other alterations	Methods	Segregation
***Ciliopathies***									
**RP-1232**	Alström	Alström	RP, hearing loss, obesity, diabetes, NPHP, hypogonadism, hypothyroidism, hyperinsulinemia, and *acantosis nigricans*	*ALMS1*	**NM_015120.4:c.4252del;** p.(Arg1418Glyfs*55)	**NM_015120.4:c.4252del;** p.(Arg1418Glyfs*55)		NGS	Y (AR)
**RP-2186**	Alström	Alström	Early RD, overweight, dilated cardiomyopathy, and diabetes	*ALMS1*	NM_015120.4:c.4477G>T; p.(Glu1493*)^61^	NM_015120.4: c.7571_7572del; p.(His2524Argfs*11)^61^		NGS	Y (AR)
**RP-2177**	Alström	Alström	CRD, hearing loss, and dilated cardiomyopathy	*ALMS1*	NM_015120.4:c.808C>T; p.(Arg270*)^31^	NM_015120.4:c.11618_11619del; p.(Ser3873Tyrfs*19)^61^		NGS	na
**RP-2069**	BBS	BBS	CRD, polydactyly, maturation and learning delay, obesity, and chronic renal failure	*IFT27*	NM_006860.4:c.104A>G; p.(Tyr35Cys)	NM_006860.4:c.350-2A>G		NGS	na
**RP-2167**	BBS	BBS	CRD, obesity, polydactyly and brachydactyly, psychomotor and learning delay, and behaviour disorder	*BBS2*	NM_031885.2:c.471G>A; Affecting 5′ splicing site^31^	NM_031885.2:c.1237C>T; p.(Arg413*)^30^		NGS	na
**RP-2228**	BBS	BBS	RP, ID, overweight since infancy, brachydactyly, chronic renal failure, and renal transplant	*BBS1*	NM_024649.4:c.1645G>T; p.(Glu549*)^33^	NM_024649.4:c.118del; p.(Cys40Alafs*2)^62^	MKKS NM_018848.3:c.724G>T; p.(Ala242Ser)^32^	chip + NGS	Y*
**RP-1814**	Joubert	Joubert	RD, nystagmus, psychomotor delay, cerebellar atrophy, chronic renal failure, and macrocephaly	*CEP290*	NM_025114.3:c.4028del; p.(Lys1343Argfs*2)^34^	NM_025114.3:c.7341dup; p.(Leu2448Thrfs*8)^35^		chip + NGS	Y (AR)
***Other specific syndromes***									
**RP-1724**	ZSSD	ZSSD	Early RP, congenital deafness, neonatal jaundice, intrahepatic biliary dysgenesis, encephalopathy, ID, hiperprolactinemia, and gynecomastia	*PEX1*	NM_000466.2:c.2528G>A; p.(Gly843Asp)^36^	NM_000466.2:c.2528G>A; p.(Gly843Asp)^36^		NGS	NA
**V-0799**	*Pseudoxanthoma elasticum*	*Pseudoxanthoma elasticum*	Macular atrophy, angioid streaks, and neovascular membrane	*ABCC6*	**NM_001171.5:c.1483_1485del;** **p.(Leu495del)**	**NM_001171.5:c.1483_1485del;** **p.(Leu495del)**		NGS	Y (AR)
***Miscellanea***									
**RP-0485**	RP + ID + deafness + congenital malformations	Usher + Koolen de Vries	RP, mild ID, hearing loss, ASD, macrocephaly, and low-set ears	*USH2A*	NM_206933.2:c.1876C>T; p.(Arg626*)^43^	NM_206933.2:c.13010C>T; p.(Thr4337Met)^44^	721Kb monosomy at 17q21.31^†^	NGS + aCGH	Y (USH2A: AR CNV: *de novo*)
**RP-1430**	RP + ID	Cohen	RP and ID	*VPS13B*	**NM_017890.4:c.1512del;** p.(Glu505Lysfs*23)	**NM_017890.4:c.1512del;** p.(Glu505Lysfs*23)		NGS	Y*
**RP-1613**	RP + neuroendocrine alteration	*OTX2*-related	LCA, congenital nystagmus, psychomotor delay, compulsive eating, muscular hypotonia, obesity, and hypogonadism	*OTX2*	**NM_172337.2:c.255** **G** **>** **A;** p.(Trp85*)			NGS	Y (*de novo*)
**RP-2140**	RP + neuroendocrine alteration	*OTX2*-related	Congenital nystagmus, early-onset CRD, developmental delay, and panhypopituitarism	*OTX2*	**NM_172337.2:c.559** **C** **>** **T;** p.(Gln187*)			NGS	Y (*de novo*)
**RP-2273**	RP + neuroendocrine alteration	BBS	Congenital nystagmus, LCA, developmental delay, congenital hypothalamic obesity, hypogonadism, *acantosis nigricans*, and clinodactyly	*CEP41*	**NM_018718.2:c.5** **C** **>** **T;** **p.(Ser2Phe)**	**NM_018718.2:c.5** **C** **>** **T;** **p.(Ser2Phe)**		NGS	na

Novel likely pathogenic variants found in this study are represented in bold. ^*^Segregation of families RP-2228 and RP-1430 could be only done in an unaffected sister and father, respectively. ^†^Monosomy at 17q21.3 (arr[GRCh37] 17q21.31(43417434_44138572)x1) encompassed the following genes: *ARHGAP27, PLEKHM1, MIR4315-1, MIR4315-2, LRRC37A4, LOC101929001, DND1P1, LOC644172, RPS26P8, CRHR1, MGC57346, SPPL2C, MAPT, MAPT-IT1, STH*, *KANSL1*. Abbreviations: aCGH: array–based comparative genomic hybridization; AR: autosomal recessive; ASD: atrial septal defect; BBS: Bardet-Biedl syndrome; CRD: cone-rod dystrophy; Dx: diagnosis; ID: intellectual disability; LCA: Leber congenital amaurosis; na: not available; NGS: next-generation sequencing; NPHP: nephronophthisis; RD: retinal dystrophy; RP: retinitis pigmentosa; Y: yes; ZSSD: Zellweger syndrome spectrum disorder.

**Table 2:** Copy Number Variants found by coverage depth analysis or array–based comparative genomic hybridization.

*Family*	*Initial diagnosis*	*Cytogenetic band*	*Rearrangement type*	*Genomic coordinates (hg19)*	*Involved genes*	*Minimun Size (kb)*	*Method*	*Validation*	*Causality*	*Additional genetic findings*
RP-0485	RP + ID + deafness + congenital malformations	17q21.31	monosomy	chr17:43417434–44138572	*ARHGAP27, PLEKHM1, MIR4315-1, MIR4315-2, LRRC37A4, LOC101929001, DND1P1, LOC644172, RPS26P8, CRHR1, MGC57346, SPPL2C, MAPT, MAPT-IT1, STH*, ***KANSL1***	721	aCGH	yes	Yes (Koolen de Vries)	Biallelic *USH2A* variants, responsible of Usher related symptoms
RP-1581	RP + ID + various disorders	11q13.2	whole gene duplication	chr11: 66277760–66300760	***BBS1***	23	NGS + aCGH	yes	VUS	in *cis with a VUS in BBS1*(maternally inherited)
RP-2009	RP + ID + various disorders	14q11.2	exon 17–19 deletion	chr14:21795786–21798554	***RPGRIP1***	2,77	NGS + MLPA	yes	Unlikely	Biallelic *SCAPER* variants^55^

aCGH: array–based comparative genomic hybridization; ID: intellectual disability; NGS: next-generation sequencing; RP: retinitis pigmentosa; VUS: variant of uncertain significance.

From the total identified variants, 23 were further considered likely causative in 14 cases based on phenotypic concordance, and also on co-occurrence with a second allele in recessive genes or the presence of *de novo* events in dominant genes (Table 1). Thus, we obtained a diagnosis rate of 30%, including known and likely disease-associated variants (Table 1 and Fig. 1). In terms of inheritance patterns, 86% of the cases had an autosomal recessive inheritance, 2 cases carried *de novo* autosomal dominant variants, and in one family, triallelism for *BBS1* and *MKKS* was suspected. Additionally, in 9 cases (19%) which carried heterozygous likely pathogenic alleles in genes responsible for recessive RD (Supplementary Table S2), no second allele was found using NGS, multiplex ligation-dependent probe amplification (MLPA), and/or array–based comparative genomic hybridization (aCGH).

### Revision of Cases with Concordant Molecular Findings and Initial Clinical Diagnosis

Our approach confirmed the initial clinical suspicion in 9 cases. Based on our first phenotypic classification, we characterized up to 70% of cases (7/10) with a clear phenotypic manifestation of ciliopathy and 40% of cases (2/5) presenting other well-defined ISRs (Fig. 1).

**Figure 2:**
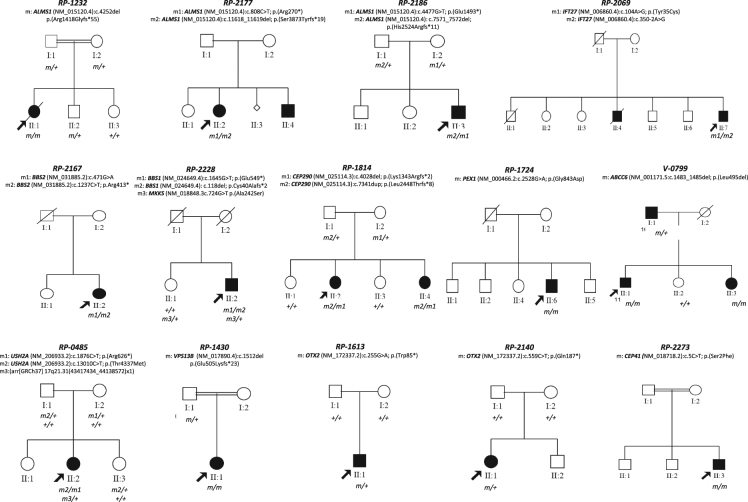
Pedigrees and co-segregation analysis for cases carrying likely disease-causing variants. Genotype of each available family member is represented below the individual symbol being “+” normal allele, and “m, m1, m2, and m3”, mutated alleles.

Three out of 4 cases with clinical suspicion of Alström syndrome carried biallelic causal *ALMS1* variants (families RP-1232, RP-2177, and RP-2186, Table 1 and Fig. 2). Similarly, 3 out of 4 of the cases previously classified as BBS carried likely pathogenic variants in known BBS-associated genes (Table 1). In family RP-2069, which comprised 2 affected siblings with RP, neurodevelopment delay, obesity, and renal alterations (Table 1 and Fig. 2), the index case was found to have two novel heterozygous likely pathogenic variants in *IFT27* (Supplementary Table S3), a splicing variant in exon 6 (c.350-2A>G), and a missense variant p.(Tyr35Cys), which affects a highly conserved residue located in a nucleotide-binding domain with GTPase activity^28^. In addition, the *IFT27*-associated phenotype is clearly consistent with the clinical presentation of our family^29^. In sporadic case RP-2167, which had characteristic BBS-associated features (Table 1), we found 2 known heterozygous *BBS2* alleles, a nonsense variant^30^, and a synonymous change that had previously been reported in homozygosis as a cause of retinitis pigmentosa^31^. This silent variant, which was located at the last nucleotide of exon 3, likely abolishes the canonical donor splice site by interfering with U1snRNA recognition. In the sporadic BBS case RP-2228, which carried 2 previously identified heterozygous variants in *MKKS*^32^ and *BBS1*^33^, a second loss-of-function (LOF) allele in *BBS1* was found by NGS analysis (Table 1). In this case, triallelism could not be completely confirmed, owing to an uninformative pedigree structure with only 1 affected patient (Fig. 2). In addition, we characterized 1 of the 2 Joubert syndrome families in our cohort. Two affected siblings presenting with early-onset RD, cerebellar atrophy, late-stage renal failure, and ID (family RP-1814, Table 1) carried previously described biallelic LOF variants in *CEP290*^34,35^ (Fig. 2).

We also confirmed the clinical diagnosis of 2 families presenting with a clear non-ciliopathy retinal syndrome (Fig. 1). The sporadic case RP-1724, who was born to an endogamous couple and presented infantile Refsum disease, homozygously carried a *PEX1* allele, p.(Gly843Asp), which is the most common causal allele in Zellweger syndrome spectrum disorders (ZSSD)^36,37^. Similar to other cases with this variant^36,37^, the patient exhibited a mild form of ZSSD with a progressive disease course. Finally, we studied an apparently pseudo-dominant family in which PXE has been suspected in 2 affected siblings and their father (V-0799, Fig. 2). All affected individuals showed a similar phenotype of angioid streaks, vision loss around 55 years, atrophic macular degeneration and dyschromatopsia. After NGS and segregation analysis, both affected siblings (II:1 and II:2) carried a novel homozygous in-frame deletion in the *ABCC6* gene, namely p.(Leu495del) (Table 1 and Fig. 2). This variant affects a phylogenetically conserved amino-acid position, where a missense change had previously been described for this disease^38^ and was predicted as deleterious by *in silico* analysis. CNV depth-read and MLPA analysis ruled out whole-gene or multi-exon deletions in the *ABCC6* gene in the family (Supplementary Fig. S2). In addition, haplotype analysis of the *ABCC6* locus and the surrounding genomic regions confirmed that an identity-by-descent (IBD) mechanism was involved in the homozygous transmission of the variant (Supplementary Fig. S2). This finding was clearly consistent with the highly endogamous ancestry of both parents. Thus, the suspected autosomal dominant transmission of this variant has been clearly ruled out in this family, accordingly to which PXE is a well recognized autosomal recessive disorder^39,40^. Therefore, the apparent pseudo-dominance in this family is most likely due to the presence of a second different pathogenic SNV in the father, that remains to be discovered after NGS sequencing. However, we cannot exclude the presence of additional non-coding variants that could explain the PXE phenotype.

### Molecular Characterization of Clinically Unclassified Cases and Refinement of Clinical Diagnosis

Five cases (11%) were phenotypically reclassified based on our molecular findings and further clinical reassessment (Table 1). Of them, 3 sporadic cases had common features of neuroendocrine disorder, psychomotor impairment, and Leber congenital amaurosis (LCA). It is noteworthy that 2 of these LCA cases (RP-1613 and RP-2140, Table 1), had diverse symptoms of hypopituitarism, and both bore *de novo* nonsense *OTX2* variants: p.(Trp85*) and p.(Gln187*), respectively (Fig. 2). LOF variants in this transcription factor were previously associated with a wide spectrum of pituitary dysfunction and congenital eye disorders ranging from anophthalmia to retinal dystrophies^41^. In a third sporadic LCA consanguineous patient (RP-2273, Table 1), an extremely rare missense variant, p.(Ser2Phe), was homozygously found in *CEP41*. This change affects a highly conserved residue in the N-terminal domain of the ciliary protein CEP41 and was predicted as damaging by *in silico* analysis (Supplementary Table S3). Although *CEP41* has previously been associated with Joubert syndrome^42^, no cerebellar abnormality was observed in the patient here reported, and his clinical picture (early-onset RD, mild ID, brachydactyly, and neuroendocrine alteration) was in principle more consistent with a BBS-like diagnosis.

**Figure 3:**
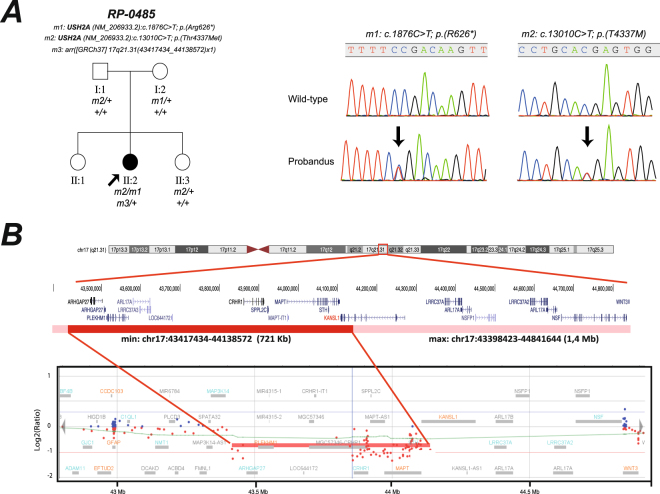
Dual diagnosis of Usher and Koolen de Vries syndromes in case index of family RP-0485 by targeted NGS and array–based comparative genomic hybridization (aCGH) (**A**). Pedigree and segregation analysis for genetic findings showed a recessive inheritance for biallelic pathogenic *USH2A* variants and a *de novo* CNV. Genotype of each available family member is represented below the individual symbol being “ + ” normal allele, and “m1, m2, and m3”, mutated alleles. Electropherograms of heterozygous carrier and wild-type individual for *USH2A* variants in exons 11 and 63 were also shown (**B**). Genomic rearrangement on 17q21.31 region identified in by aCGH. Chromosome and gene views of the affected chromosome show an aberrant deletion of minimum size of 721Kb (genomic coordinates: chr17:43,417,434-44,138,572) and maximum ~1,4 Mb (chr17:43,398,423-44,841,644), which is highlighted in dark and light red colour, respectively. An abnormal pattern of one copy was observed for 80 probes covering 16 genes (*ARHGAP27, PLEKHM1, MIR4315-1, MIR4315-2, LRRC37A4, LOC101929001, DND1P1, LOC644172, RPS26P8, CRHR1, MGC57346, SPPL2C, MAPT, MAPT-IT1, STH*, *KANSL1*). The vertical axis shows the position along the genome (hg19) and the horizontal axis the log2 intensity ratio values (−2/−1: deletions, 0: normal pattern, + 1/ + 2: duplications).

In the sporadic case RP-0485, who had RD, neurosensorial hypoacusia, mild ID, and several congenital malformations (Fig. 3 and Table 1), the co-occurrence of 2 rare genetic disorders was identified. First, NGS analysis identified 2 biallelic *USH2A* variants, both of which had previously been associated with Usher syndrome^43,44^, that were responsible for the retinal and hearing manifestations. Further high-resolution aCGH analysis enabled us to identify a 721-kb *de novo* microdeletion at 17q21.31 (Fig. 3), including the *KANSL1* gene, which had been reported to cause Koolen-de Vries syndrome^45^. Finally, in a consanguineous Middle-Eastern patient (RP-1430) with RD and mental disability, a novel causative homozygous frameshift variant was found in the *VPS13B* gene, which is associated with Cohen syndrome. Unfortunately, a more accurate diagnosis could not be established, since few clinical data were available, and clinical re-evaluation of this patient could not be performed.

## Discussion

ISRs comprise a diverse spectrum of highly heterogeneous and overlapping clinical conditions. Clinical diagnosis is often challenging, as some of the characteristic systemic features may not develop until later in infancy and adulthood or may even evolve and change over time. Several multigene panel approaches have been used for molecular characterization of specific retinal ciliopathies, such as BBS, Joubert syndrome, and Usher syndrome, yielding variable diagnostic rates^7,18,19,46,47^. Here, we first studied a more heterogeneous and large cohort of “non-naive”, i.e. previously screened ISR patients, who were all recruited in a single center, and were similar to those found in daily clinical practice. We included not only cases with a clear suspicion of syndromic forms of retinal disease, but also atypical cases that did not exactly fit any known ISR.

Our findings confirm the clinical utility of targeted NGS strategies, demonstrating an overall diagnostic rate of 30%, which is comparable with those obtained from similar studies using prescreened RD patients^25,48^. As expected from the heterogeneity of the cases included, the diagnostic yield obtained in the present ISR cohort was not uniform. While we obtained a high mutation detection rate (70%) in patients with clinically suspected specific ciliopathies, similar to previous studies^46,47^, the diagnostic rate decreased in patients with unspecific symptoms of ISR. Notably, the responsable molecular mechanism was identified in at least 5 of these patients, thus our study highlighted the potential of NGS to improve diagnostic accuracy in complex phenotypes. In this sense, the panel led us to clinically characterize 2 sporadic cases in which LCA was accompanied by neuroendocrine symptoms as bearing *de novo OTX2* variants. The clinical symptoms of both cases overlapped with the previously *OTX2*-associated phenotypes, including pituitary dysfunction and retinal dystrophy^49^. Consequently, both patients were also genetically reclassified as having a condition based on dominant inheritance. The identification of *de novo* events highlights the relevance of obtaining a correct genetic diagnosis in sporadic genetic cases as first step to enable a more accurate genetic counseling^50^.

We were able to reclassify a miscellaneous case within the ciliopathy group owing to the identification of a novel homozygous likely causal *CEP41* variant. Although this ciliary gene was previously associated with Joubert syndrome^42^, this sporadic case had RP with neuroendocrine symptoms that seemed to be more compatible with a BBS-like form. In addition, the normal brain MRI scan in this patient excluded Joubert syndrome as clinical diagnosis. Furthermore, we found biallelic pathogenic variants in the *IFT27* gene in a BBS patient. To our knowledge, this is the second case of BBS bearing *IFT27* alterations in the literature^29^.

In 9 cases, we found only a monoallelic likely pathogenic variant in a well-known recessive gene. The presence of a second pathogenic variant was not fully excluded, as it might reside in deep intronic, regulatory, and even highly repetitive regions that were not targeted in our gene panel. The clinical presentation in most cases was reasonably consistent with the phenotypic features of the suspected gene. For instance, 3 of 11 ciliopathy-like patients carried heterozygous pathogenic variants in ciliopathy genes, such as *ALSM1*, *USH2A*, and *WDPCP*, thus it might support the initial clinical suspicion. Therefore, some of these inconclusive genetic findings could also be helpful to guide further management and follow-up of these patients. However, additional molecular analysis of the above-mentioned unexplored regions should be performed using custom targeted resequencing or even whole-genome sequencing (WGS).

Structural variants also represent an underestimated mutational burden that contributes to missing heritability and oligogenic mechanisms, not only in non-syndromic RD, but also in BBS and Joubert syndrome^20,24–26,51,52^. Here, we report novel heterozygous multi-exon structural variants for *BBS1* and *RPGRIP1* that were identified by targeted comparative read-depth analysis. However, their possible causality is unclear, since no second pathogenic allele was found in *trans* in these genes. Therefore, additional studies would be necessary to determine whether the variants represent random background variation or contribute to the disease. Nonetheless, our results also suggest that CNV and re-sequencing studies are both required to fully delineate the complexity of disease architecture in ISR phenotypes.

The low diagnostic rate found in cases with suspected atypical ciliopathies or complex presentations also suggests the participation of additional genes not present in the panel, whether novel candidate genes or genes recently associated with ISRs (e.g. *CEP120* or *GRID2*)^53,54^. Further whole-exome sequencing (WES) or WGS could prove useful for characterization of additional patients in our cohort. In this sense, biallelic pathogenic variants were identified in *SCAPER*, a novel RD gene explaining RP with ID, in 2 uncharacterized cases of the miscellaneous group, as we recently published^55^. An additional plausible mechanism that could explain some negative cases with a complex phenotypic spectrum is the possibility of multiple clinical diagnoses due to the co-existence of molecular defects in several disease-causing genes. Dual molecular diagnosis was recently reported in at least 4% of cases with informative WES^22,56^. Here, we observed co-occurrence of Usher syndrome and Koolen de Vries syndrome, each segregating independently as recessive and *de novo* events, respectively. Biallelic *USH2A* variants, first identified by NGS, were responsible for RP and neurosensorial hypoacusia, but they do not completely explain the phenotypic complexity of this case. Other features, such as mild ID, atrial septal defect, macrocephaly and low-set ears, were caused by a coexistent *de novo 17q21.31* microdeletion, including the *KANSL1* gene, which is associated with Koolen-de Vries syndrome^45^. Thus, further NGS testing of non-syndromic associated RD genes in combination with aCGH analysis may prove very useful for ruling out the co-existence of several genetic events in uncharacterized atypical cases^22^.

Our study highlights the huge potential of genetic testing to obtain prompt molecular diagnoses in complex ISR cases using relatively low-cost, robust, accurate, and clinically useful multigene NGS panels and CNV analysis. Thus, this combined strategy increased our understanding of well-defined phenotypes of ISR, such as retinal ciliopathies. Implementation in routine clinical practice of similar approaches based on NGS panels with a wider gene spectrum including not only reported RD and ciliary genes, but also genes associated with other retinal conditions, would reduce the time and cost of diagnosing ISRs and provide an earlier diagnosis of systemic features, even before they appear. This is especially important for clinical follow-up in medically actionable diseases and also for enrollment of patients in future gene therapy–based treatments. Alternatively, clinical or WES seems to be a very useful choice for cases presenting with more unspecific or widely complex ISR phenotypes and could potentially improve molecular diagnostic yields.

## Materials/Subjects and Methods

### Clinical diagnosis and sample collection

We selected genetically non-characterized patients with syndromic retinopathies from our database of RD patients who attended Fundación Jiménez Díaz University Hospital (FJD, Madrid, Spain) in the last 25 years. We excluded typical Usher syndrome patients, as they were already assessed using a specific NGS Usher gene panel^47^. The clinical diagnosis was made according to previously established criteria and included ophthalmic, physical, and additional examinations^10,12,14,57,58^. The cohort included cases with various suspected patterns of inheritance, including autosomal recessive, autosomal dominant, and sporadic cases. This study was reviewed and approved by the FJD Ethics Committee and was performed according to the tenets of the Declaration of Helsinki and its reviews. The participants, or their legal guardians, signed a written informed consent form before the study. DNA samples were collected from FJD Biobank.

### Clinical Genetic Testing

Following routine clinical procedures, all patients had previously been screened using commercial genotyping microarrays (AsperBiotech, Tartu, Estonia) for previously known pathogenic variants in several genes associated with BBS, Alström syndrome, LCA, or autosomal recessive retinitis pigmentosa. In cases compatible with BBS or Alström syndrome, Sanger-sequencing was performed to screen for the most prevalent mutated exons and/or genes, as previously reported^57,58^. Homozygosity mapping studies were also performed in some families^57,59^.

### Target Enrichment and Bioinformatic Analysis

#### Gene Panel Design, Library Construction, and Sequencing

A custom targeted NGS approach was designed to specifically study ISR patients using the Nextera Rapid Capture kit (Illumina, Cambridge, UK) for target enrichment. The DesignStudio software tool (Illumina) was used to design the gene panel. We selected genes based on previous associations with ISRs, including 86 genes reported in the Retnet database (data accession in 2015) and 17 genes after a thorough revision of literature. In addition, 18 very likely candidate genes were also included due to their previously involvement in other non-syndromic ciliopathies.

Many of them show a high degree of phenotypic overlap and appear to be functionally related (Supplementary Fig. S1). The complete list of genes targeted is detailed in Supplementary Table S1 and includes the following: i) 71 ciliopathy-related genes accounting for RD and multisystemic disease; ii) 18 ciliary genes previously implicated in non-syndromic RD or associated with non-retinal ciliopathies, such as isolated nephronophthisis, were also added as likely candidate genes; and iii) 32 non-ciliopathy genes including PBDs and lysosomal disorders (5 genes), syndromic vitreoretinopathies (7 genes), and a miscellanea of syndromic retinal alterations with overlapping systemic features (20 genes). The gene panel included coding and non-coding exons and targeted flanking upstream and downstream 50-bp and 5′ and 3′ UTR regions and also known deep intronic variants previously identified in the *BBS1, CEP290, OFD1, and USH2A* genes (Supplementary Table S4). In total, the library contained 2,459 target regions covered by 3,813 probes spanning 577 kb.

Libraries for 47 samples were prepared following the manufacturer’s protocol. Briefly, DNA samples were quantified using fluorometric assays (PicoGreen, Invitrogen, Thermo Fisher Scientific, MA, USA) and normalized to prepare 50 ng of starting material at 5 ng/µl. Samples were tagmented, PCR amplified, and cleaned up. Libraries were pooled with 24 samples, multiplexed, hybridized for 90 min, and captured. A second round of hybridization, capture, PCR amplification, and clean-up was performed. Final libraries were quantified using qPCR with the KAPA Library Quantification Kit (Kapa Biosystems, London, UK) and validated by capillary electrophoresis using a 2100 Bionalyzer (Agilent Technologies, Santa Clara, CA, USA). Finally, 1.8 pm of 24 equimolecularly pooled libraries were loaded into a NextSeq500 flow cell and sequenced using the NextSeq500 Mid Output reagents Kit v1 (Illumina, Cambridge, UK) to obtain 150-bp paired-end reads. The sequencing had an average coverage of 435X with approximately 85% reads on target, and 93% of target regions of these 121 genes were completely covered (Supplementary Fig. S3).

The affected father (I:1) from family V-0799 has also been analyzed using targeted clinical exome. Libraries were prepared using TruSightOne (Illumina) following the manufacturer’s protocol. The captured libraries were sequenced with Illumina NextSeq500 with 150-bp paired-end reads.

#### Bioinformatic Analyses

A specific custom pipeline for Illumina Nextera technology implemented on the commercial DNAnexus platform (https://www.dnanexus.com/) was used for the bioinformatics analysis as previously described^25,48^. Briefly, Prinseq-lite v0.20.3 was used to calculate read statistics, and BWA-MEM v0.7.5a with default parameters was used to map the reads. Samtools v0.19 was then used to sort and index the BAM files, and Picard MarkDuplicates v1.119 was used to mark duplicates. Freebayes v9.9.13 was used for variant calling, Picard CalculateHSMetrics v1.119 to calculate metrics, and BedTools and custom code to determine minimum coverage. Identified variants were annotated using GATK v2.4. Gene coverage was calculated using BedTools to extract the number of reads overlapping any genome region from the BAM file of each sample. From this, coverage was calculated per gene and sample by counting the reads per nucleotide inside the gene coordinates. Regions with a coverage of fewer than 10 reads were excluded.

To verify the reliability of our targeted NGS approach, we used a validation cohort consisting of 5 out of the total 47 patients analyzed, which carried 6 different heterozygous variants previously detected by genotyping microarray (Supplementary Table S5). All variants used as controls were correctly identified.

A CNV analysis was performed with NGS data following the previously reported CoNVaDING method, which had been specifically developed for gene panel data at high-coverage and enables accurately single-exon detection^60^. Briefly, it uses the average coverage of each specified target to perform calculations starting from BAM files. Then, it selects a group of control samples with the coverage pattern that is most similar to that of the sample to be analyzed, which are used in the normalization step. Finally, it performs a filtering step that renders the list with deletions and duplications. The method includes QC metrics that make it possible to distinguish high-quality from low-quality samples^60^.

#### Variant Prioritization and Classification

Potentially pathogenic variants were sequentially assessed as previously described^25,48^, based on the following criteria: i) variants previously reported as pathogenic in mutation databases, including HGMD Professional (http://www.biobase-international.com/product/hgmd) and the Leiden Open Variation Database (http://www.lovd.nl/3.0/home); ii) LOF variants such as nonsense, frameshift, and those located at the canonical splice site; iii) variants filtered out, focusing on rare variants with a minor allele frequency (MAF) ≤0.005 in 1000 Genomes (http://www.ncbi.nlm.nih.gov/variation/tools/1000genomes/), Exome Variant Server (EVS) (http://evs.gs.washington.edu/EVS/), ExAC Browser (http://exac.broadinstitute.org/), and CIBERER Spanish Variant Server (http://csvs.babelomics.org/); iv) missense SNV and in-frame deletions that induced an alteration in the protein predicted as pathogenic in at least 2 of the *in silico* tools used to predict pathogenicity (SIFT, Polyphen2, Mutation Taster, Align GVGD, PROVEAN); v) non-canonical splicing variants *in silico* predicted to cause a deleterious effect by several splicing tools (Human Splicing Finder, MaxEntScan, Splice Site Finder-like, NNSPLICE, GeneSplicer, ESEFinder); vi) genes associated with the most likely clinical phenotype in each case. Alamut software (Interactive Biosoftware, Rouen, France) was used to infer *in silico* predictions of variant pathogenicity.

### Validation Studies

Sequence variants of interest identified by NGS were verified and segregated in the respective families by Sanger sequencing following PCR amplification of the respective coding exons and adjacent intronic sequences by standard protocols (primers are available on request).

### Multiplex Ligation-dependent Probe Amplification (MLPA) analysis

MLPA was performed according to manufacturer instructions using commercially available kits (MRC-Holland, Amsterdam, Netherlands). The *RPGRIP1* deletion on exons 17–19 was validated using MLPA SALSA p222 covering exons 18 and 19. The MLPA SALSA P092 was used to rule out CNVs in the *ABCC6* gene. The amplified fragments were separated by capillary electrophoresis through an ABI 3130xl automatic analyzer (Applied Biosystems) and analyzed using GeneMapper (Applied Biosystems) and Coffalyser (MRC-Holland) software programs.

### Array–based comparative genomic hybridization

Contiguous gene deletion syndromes was assessed by means of aCGH in 14 patients with atypical complex phenotypes in which RD was accompanied by ID and/or congenital malformations. The aCGH analysis was performed using the Agilent SurePrint G3 CGH + SNP Microarray Kit 2 × 400 K (Agilent Technologies, Santa Clara, CA, USA), which contains 292 097 CGH probes and 119 091 SNP probes with median spacing of 7.2 kb. The CGH probes are gene- and exon-biased, focusing coverage on regions of clinical interest in the genome. Briefly, genomic DNA (500 ng) from the patient and from a sex-matched control were labeled with Cy3-dUTP and Cy5-dUTP fluorochromes using the Sure Tag DNA Labeling Kit (Agilent Technologies), and further hybridized on microarray according to Agilent protocols. The slide was scanned on a SureScan G4 900DA scanner (Agilent Technologies). TIFF image was analyzed using Agilent CytoGenomics v.2.7 based.

### Haplotype Analysis

Five polymorphic microsatellite markers on 16p13.13-p12.1 with high heterozygosity (D16S3075, D16S3103, D16S3046, D16S420 and D16S3068) flanking around 13 Mb (chr16:12,115,341-25,549,627) of the *ABCC6* gene were used to investigate the IBD mechanism in the family V-0779. For the STR genotyping, PCR products were analyzed using the automated ABI 3130xl Genetic Analyzer (Applied Biosystems) and further analyzed with the GeneMapper v3.5 software (Applied Biosystems). Haplotype reconstruction was performed using the software Cyrillic ver. 2.1 (Cyrillic Software, Wallingford, UK).

## Electronic supplementary material

Supplementary Figures and Tables
