# Supplementary material for: Combining targeted panel-based resequencing and copy-number variation analysis for the diagnosis of inherited syndromic retinopathies and associated ciliopathies
Source: Sci Rep. 2018 Mar 27;8:5285. doi: 10.1038/s41598-018-23520-1 (PMC5869593; doi:10.1038/s41598-018-23520-1)
Supplement: Supplementary file 1 — Supplementary Figures and Tables [file 41598_2018_23520_MOESM1_ESM.pdf]

“Combining targeted panel-based resequencing and copy-number variation analysis for the diagnosis of inherited syndromic retinopathies and associated ciliopathies“

Iker Sanchez-Navarro* [1, 2] , Luciana Rodrigues Jacy da Silva* [1, 3], Fiona Blanco-Kelly [1, 2], Olga Zurita [1, 2], Noelia Sanchez-Bolivar [1], Cristina Villaverde [1,2], Maria Isabel Lopez-Molina [4], Blanca Garcia-Sandoval [4], Saoud Tahsin Swafiri [1], Pablo Mínguez [1], Rosa Riveiro-Alvarez [1, 2], Isabel Lorda [1, 2], Rocío Sanchez-Alcudia [1, 2], Raquel Perez-Carro [1], Diana Valverde [5], Yichuan Liu [6,7], Lifeng Tian [6], Hakon Hakonarson [6, 8, 9], Almudena Avila-Fernandez [1, 2], Marta Corton* [1, 2], Carmen Ayuso* [1, 2].

[1] Department of Genetics, Instituto de Investigaciones Sanitarias – Fundacion Jiménez Díaz University Hospital (IIS-FJD-UAM) Madrid, Spain.

[2] Centre for Biomedical Network Research on Rare Diseases (CIBERER), ISCIII, Madrid, Spain.

[3] Universidade de Mogi das Cruzes, São Paulo, Brazil.

[4] Department of Ophthalmology, Fundacion Jiménez Díaz University Hospital, Madrid, Spain.

[5] Department of Biochemistry, Genetics and Immunology, Faculty of Biology, Universidad de Vigo, Vigo, Spain.

[6] Center for Applied Genomics, Abramson Pediatric Research Center, The Children's Hospital of Philadelphia, Philadelphia, PA, USA.

[7] Medical Scientist Training Program, Perelman School of Medicine, University of Pennsylvania, Philadelphia, PA, USA.

[8] Division of Human Genetics, Children's Hospital of Philadelphia, Philadelphia, PA, USA.

[9] The Perelman School of Medicine, University of Pennsylvania, Philadelphia, PA, USA.

*These authors have contributed equally

Correspondence:

Carmen Ayuso, MD, PhD & Marta Corton, PhD

Department of Genetics

Fundación Jiménez Díaz University Hospital

Av. Reyes Católicos nº 2.

28040 Madrid, Spain

cayuso@fjd.es / mcorton@fjd.es

Phone ##34 609612728

SUPPLEMENTARY FIGURE LEGENDS

Supplementary Figure S1. Network representation of 121 disease-associated genes involved in inherited syndromic retinopathies and/or non-retinal ciliopathy. Genes from the panel are classified in five disease classes: i) Retinal systemic ciliopathies (dark blue), ii) other ciliary genes (non-syndromic retinal dystrophies and non-retinal ciliopathy, light blue), iii) syndromic vitreoretinopathies (green), iv) peroxisomal disorders (yellow) and v) a group of genes associated with miscellaneous syndromes (red). The links between the genes represent two types of relationships, functional and phenotypic. To simplify the representation, dark connections indicate genes having both types of associations and light connections one of the two. Functional associations are taken from STRING database v10 (combined score >= 400). Phenotypic associations represent genes sharing a significant number of phenotypes (Human Phenotype Ontology (HPO) annotation). The significant overlap of HPO terms is calculated as follow: i) genes are annotated with their HPO terms removing very common or functional unrelated terms ("Clinical modifier" terms, except the ones under "Triggered by", "Constitutional symptom" and all under the classes "Frequency" and "Mode of inheritance" and "Mortality/Aging"), ii) calculate a score for each possible interaction (all pairs of genes in the human genome) as the percentage of overlap of annotated HPO terms taking as 100% the unique HPOs from both genes, iii) all scores are normalized to calculate a z-score for each interaction (z-score = (score - mean) / stddev), iv) z-score values > 1.96 are taken as significant.

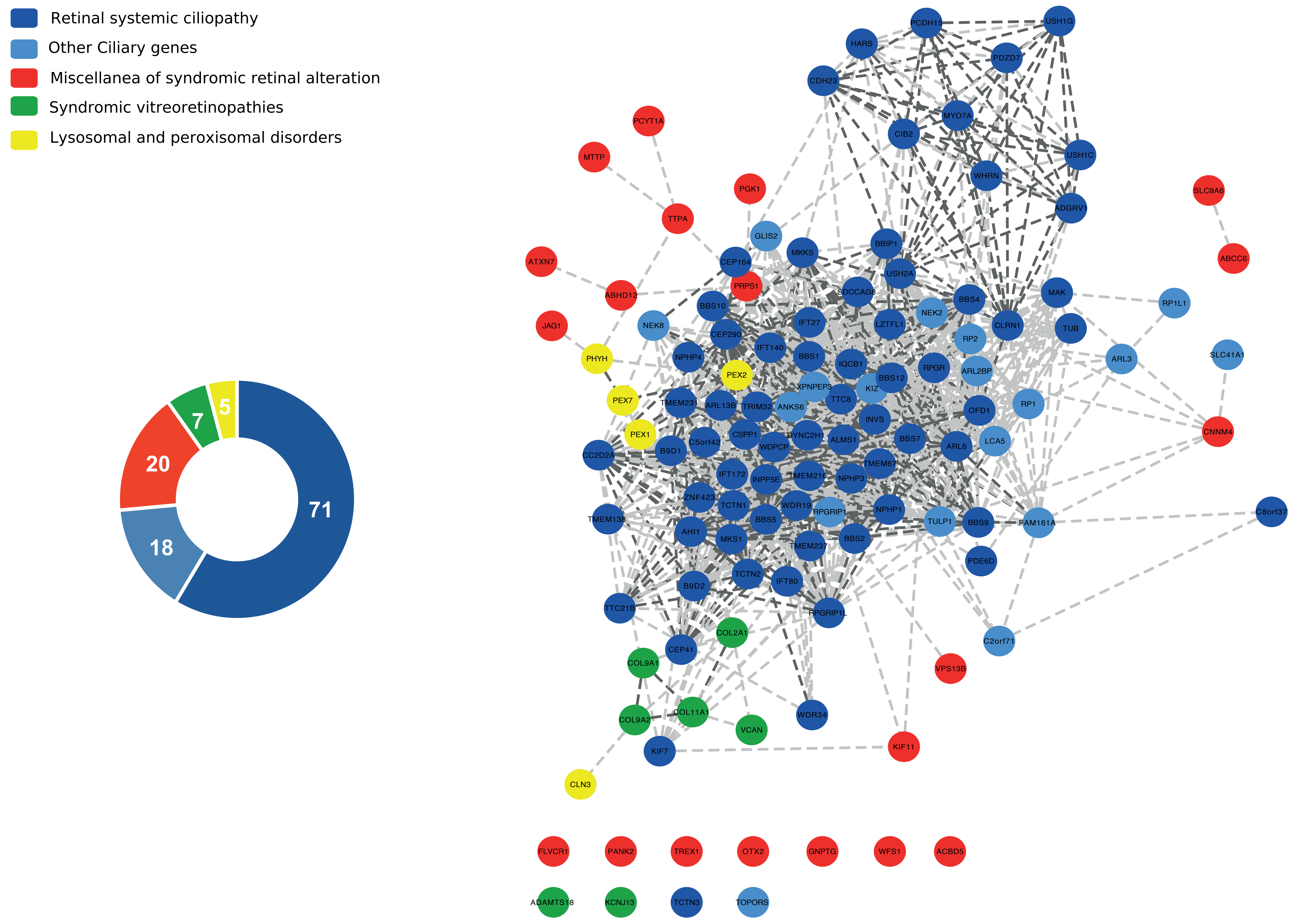

Retinal systemic ciliopathy
Other Ciliary genes
Miscellanea of syndromic retinal alteration
Syndromic vitreoretinopathies
Lysosomal and peroxisomal disorders
71
18
20
7
5
PCDH15
USH1G
HARS
PDZD7
CDH23
MYO7A
CIB2
USH1C
WHRN
ADGRV1
PCYT1A
MTTP
PGK1
TTPA
SLC9A6
GLIS2
MKKS
BBIP1
CEP164
ATXN7
USH2A
ABCC6
PRPS1
ABHD12
SDCCAG8
BBS4
MAK
RP1L1
BBS10
NEK2
JAG1
NEK8
IFT27
LZTFL1
CLRN1
TUB
CEP290
RP2
PHYH
NPHP4
IFT140
BBS1
IQCB1
RPGR
ARL2BP
ARL3
SLC41A1
PEX2
BBS12
XPNPEP3
KIZ
PEX7
TMEM231
ARL13B
TRIM32
ANKS6
TTC8
OFD1
RP1
INVS
PEX1
CSPP1
DYNC2H1
ALMS1
BBS7
CNNM4
C5orf42
CC2D2A
B9D1
WDPCP
ARL6
LCA5
TMEM67
IFT172
INPP5E
TMEM216
NPHP3
ZNF423
TCTN1
WDR19
RPGRIP1
NPHP1
C8orf37
TMEM138
BBS5
TULP1
BBS9
FAM161A
AHI1
MKS1
TMEM237
BBS2
PDE6D
TCTN2
IFT80
B9D2
TTC21B
RPGRIP1L
COL2A1
C2orf71
CEP41
COL9A1
VPS13B
COL11A1
COL9A2
VCAN
WDR34
KIF11
KIF7
CLN3
FLVCR1
PANK2
TREX1
OTX2
GNPTG
WFS1
ACBD5
ADAMTS18
KCNJ13
TCTN3
TOPORS

Supplementary Figure S2. Additional genetic analysis carried out in family V-0799. A. MLPA analysis of *ABCC6* in the both affected siblings (II:1 and II:2) ruling out whole-gene or exon 12 deletions. The data were interpreted by taking the ratio of each probe signal between the patient and references DNA samples. A ratio of 1.0 indicates the presence of 2 alleles (normal diploid), while a ratio below 0.6 or above 1.4 suggests deletion or duplication of the target sequence, respectively. B. Haplotype analysis of the *ABCC6* locus and the neighbors genomic regions on 16p. Haplotypes were constructed using genotypes from five informative polymorphic microsatellites on 16p13.13-p12.1. Genotype for the variant (c.1483_1485del) in *ABCC6* was also represented. Inferred genotypes for deceased mother (I:2) is represented in brackets. An identity-by-descent (IBD) region of 13 Mb was shared by both affected siblings.

A

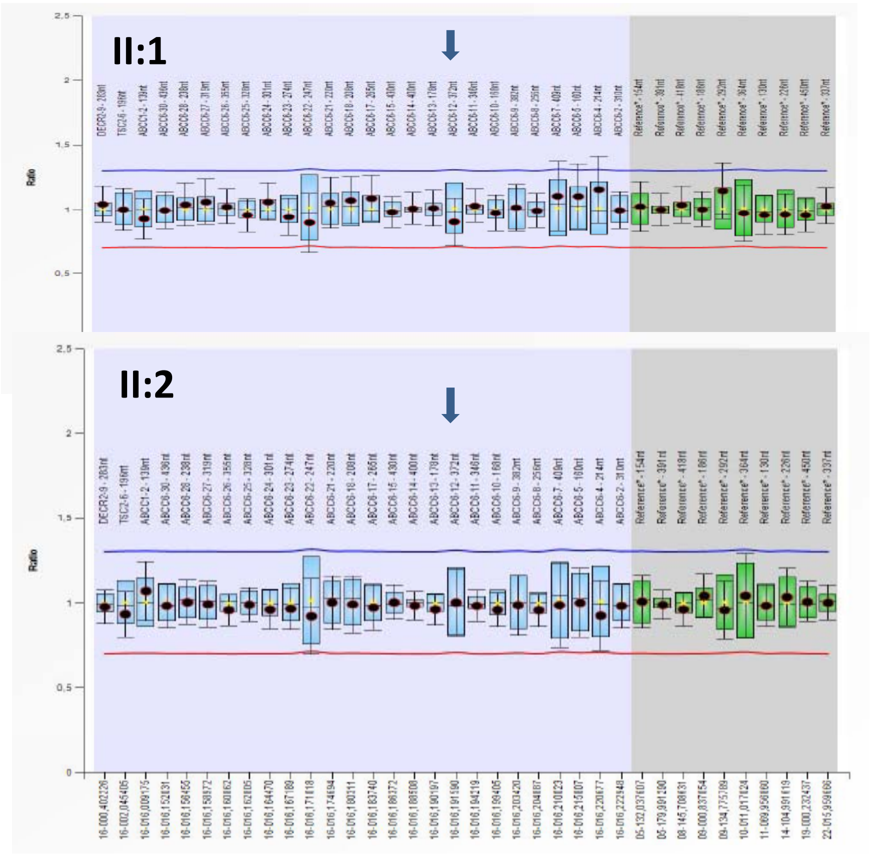

B

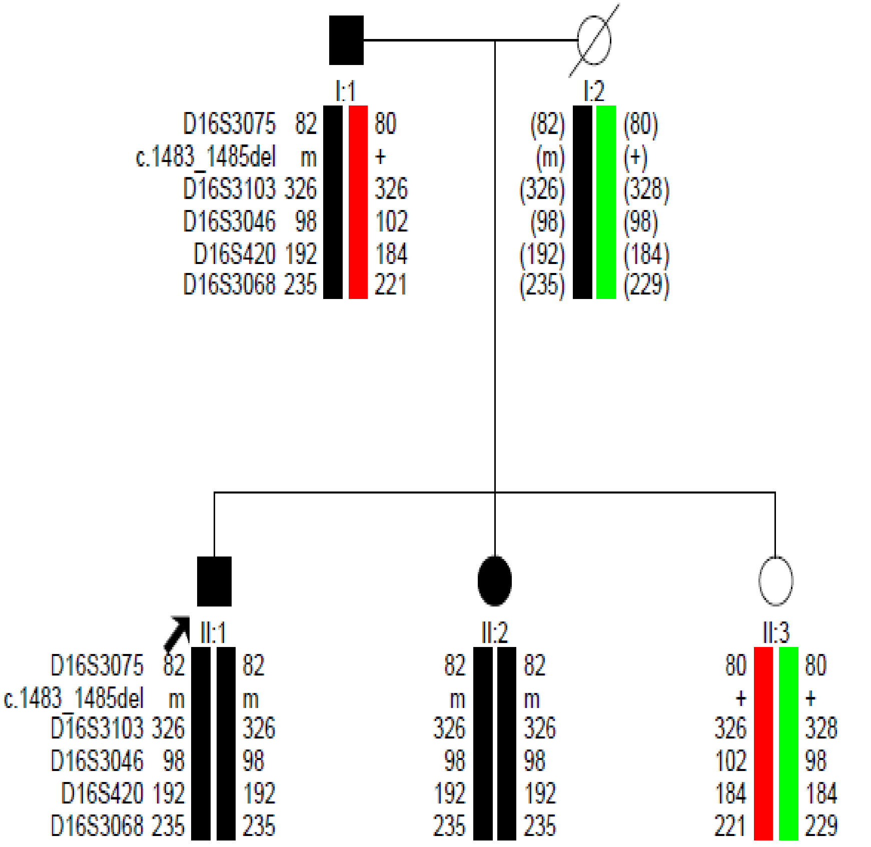

Marker	Chr Band	Position (Mb)
**D16S3075**	16p13.13	12.1
ABCC6	16p13.11	16.1
**D16S3103**	16p12.3	17.4
**D16S3046**	16p12.3	20.9
**D16S420**	16p12.1	24.2
**D16S3068**	16p12.1	25.5

Supplementary Figure S3. Representation of the mean coverage for the 121 target genes in all 47 samples. For every gene, mean read number was calculated for all the samples and represented as boxplots. Except for certain highly-repetitive regions unevenly covered, most of the genes studied in the panel approach the global mean coverage of 435x. Dots represent outlier values from the distribution.

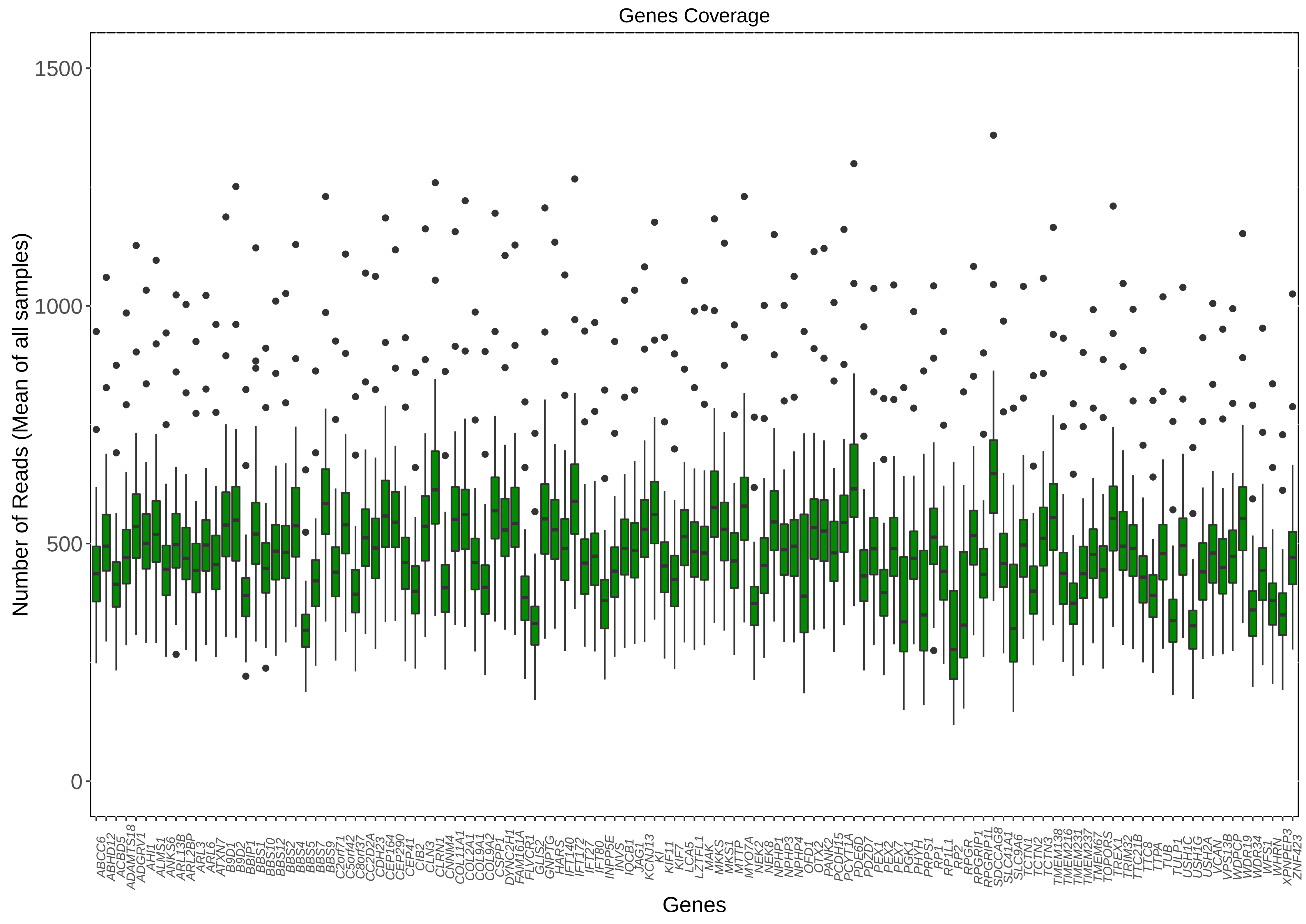

Genes Coverage
Number of Reads (Mean of all samples)
1500
1000
500
0
ABCC6
ABHD12
ACBD5
ADAMTS18
ADGRV1
AHI1
ALMS1
ANKS6
ARL13B
ARL2BP
ARL3
ARL6
ATXN7
B9D1
B9D2
BBIP1
BBS1
BBS10
BBS12
BBS2
BBS4
BBS5
BBS7
BBS9
C2orf71
C5orf42
C8orf37
CC2D2A
CDH23
CEP164
CEP290
CEP41
CIB2
CLN3
CLRN1
CNNM4
COL11A1
COL2A1
COL9A1
COL9A2
CSPP1
DYNC2H1
FAM161A
FLVCR1
GLIS2
GNPTG
HARS
IFT140
IFT172
IFT27
IFT80
INPP5E
INVS
IQCB1
JAG1
KCNJ13
KI
KIF11
KIF7
LCA5
LZTFL1
MAK
MKKS
MKS1
MTTP
MYO7A
NEK2
NEK8
NPHP1
NPHP3
NPHP4
OFD1
OTX2
PANK2
PCDH15
PCYT1A
PDE6D
PDZD7
PEX1
PEX2
PEX7
PGK1
PHYH
PRPS1
RP1
RP1L1
RP2
RPGR
RPGRIP1
RPGRIP1L
SDCCAG8
SLC41A1
SLC9A6
TCTN1
TCTN2
TCTN3
TMEM138
TMEM216
TMEM231
TMEM237
TMEM67
TOPORS
TREX1
TRIM32
TTC21B
TTC8
TTPA
TUB
TULP1
USH1C
USH1G
USH2A
VCAN
VPS13B
WDPCP
WDR19
WDR34
WFS1
WHRN
XPNPEP3
ZNF423
Genes

Supplementary Table S1. Genes included in the present study.

Gene	Chromosome	Start position	End Position	No. Exons	Inheritance	Disease
Retinal systemic ciliopathy						
ADGRV1	5	89854567	90460083	91	AR	Usher
AHI1	6	135605060	135818953	30	AR	Joubert
ALMS1	2	73612836	73837096	23	AR	Alstrom
ARL13B	3	93698933	93774572	13	AR	Joubert
ARL6	3	97483545	97517423	10	AR	BBS
B9D1	17	19240817	19281545	11	AR	MKS // Joubert
B9D2	19	41860272	41870128	4	AR	MKS // NPHP
BBIP1	10	112658438	112679174	6	AR	BBS
BBS1	11	66278069	66301134	17	AR	BBS
BBS10	12	76738216	76742272	2	AR	BBS
BBS12	4	123653807	123666148	3	AR	BBS
BBS2	16	56518209	56554058	17	AR	BBS
BBS4	15	72978470	73030867	19	AR	BBS
BBS5	2	170335956	170363215	12	AR	BBS
BBS7	4	122745434	122791702	20	AR	BBS
BBS9	7	33169102	33645730	23	AR	BBS
C5orf42	5	37106280	37249580	52	AR	Joubert
C8orf37	8	96257091	96281512	6	AR	BBS // non-syndromic RP
CC2D2A	4	15471439	15603230	40	AR	Joubert // RP with ID
CDH23	10	73156641	73575754	74	AR	Usher
CEP164	11	117192444	117284032	36	AR	Senior-Løcken
CEP290	12	88442740	88536043	54	AR	BBS// Joubert // Senior- Løcken // MKKS // non-syndromic LCA
CEP41	7	130033562	130081101	13	AR	Joubert
CIB2	15	78396898	78423927	6	AR	Usher
CLRN1	3	150643900	150690836	9	AR	Usher
CSPP1	8	67976553	68108899	29	AR	Joubert // Jeune
DYNC2H1	11	102980110	103350641	90	AR	Jeune
HARS	5	140053440	140071362	14	AR	Usher
IFT140	16	1560378	1662159	31	AR	Mainzer-Saldino
IFT172	2	27667190	27712621	48	AR	BBS// Jeune // Mainzer-Saldino // non-syndromic RP
IFT27	22	37154196	37172227	9	AR	BBS
IFT80	3	159974724	160117370	24	AR	Jeune
INPP5E	9	139323017	139334306	10	AR	Joubert // MORM

Abreviations: AD: autosomal dominant; AR: autosomal recessive; XL: X-linked; BBS : Bardet-Biedl syndrome; CRD: cone-rod dystrophy; HARP: hypoprebetalipoproteinemia, acanthocytosis, retinitis pigmentosa, palladial degeneration; LCA: Leber congenital amaurosis; MKS: Meckel syndrome; MKKS: McKusick-Kaufman syndrome; MORM: Mental retardation, truncal obesity, retinal dystrophy and micropenis; NPHP: nephronophthisis, PHARC: polyneuropathy, hypoacusia, ataxia, retinitis pigmentosa, and cataracts; ID : intellectual disability; RD: retinal dystrophy; RP: retinitis pigmentosa, S.: Syndrome, ZSSD: Zellweger syndrome spectrum disorders. Chromosome position are based on human genome build hg19.

Supplementary Table S1. Genes included in the present study (Continued).

Gene	Chromosome	Start position	End Position	No. Exons	Inheritance	Disease
Retinal systemic ciliopathy (continued)						
INVS	9	102861452	103063476	20	AR	Senior- Løocken // NPHP
IQCB1	3	121488560	121553976	15	AR	Senior-Løocken
KIF7	15	90171151	90198732	19	AR	Joubert
LZTFL1	3	45864760	45957266	13	AR	BBS
MAK	6	10762906	10838838	16	AR	BBS // MKKS
MKKS	20	10385378	10414937	7	AR	BBS // MKKS
MKS1	17	56282747	56297016	19	AR	BBS // MKKS
MYO7A	11	76839260	76926336	53	AR	Usher
NPHP1	2	110880864	110962689	22	AR	Senior-Løocken// Joubert
NPHP3	3	132399403	132441353	27	AR	Senior-Løocken
NPHP4	1	5922820	6052583	30	AR	Jeune
OFD1	X	13752782	13787530	23	XL	Joubert // orofaciodigital S.// Simpson-Golabi-Behmel S. // non-syndromic RP
PCDH15	10	55562483	56561101	43	AR	Usher
PDE6D	2	232597097	232646024	5	AR	Joubert
PDZD7	10	102767390	102790964	18	AR	Usher
RPGR	X	38128373	38186838	20	XL	Non-syndromic RP and primary ciliary dyskinesia
RPGRIP1L	16	53633768	53737821	27	AR	Joubert // MKKS
SDCCAG8	1	243419257	243663443	18	AR	Senior-Løocken // BBS // NPHP
TCTN1	12	111051782	111086985	20	AR	Joubert
TCTN2	12	124155610	124193000	19	AR	MKKS
TCTN3	10	97423103	97453950	14	AR	Joubert // Mohr-Majewski S.
TMEM138	11	61129423	61137025	7	AR	Joubert
TMEM216	11	61159782	61166385	7	AR	Joubert // MKKS
TMEM231	16	75571965	75590234	10	AR	Joubert
TMEM237	2	202484857	202508302	13	AR	Joubert
TMEM67	8	94767022	94831510	34	AR	Joubert // MKKS// BBS // NPHP
TRIM32	9	119449531	119463629	3	AR	BBS
TTC21B	2	166729822	166810398	29	AR	Jeune
TTC8	14	89290928	89344385	15	AR	BBS // non-syndromic RP
TUB	11	8060130	8127704	14	AR	RD with Obesity
USH1C	11	17515392	17566013	28	AR	Usher
USH1G	17	72912126	72919401	3	AR	Usher
USH2A	1	215796186	216596788	73	AR	Usher
WDPCP	2	63348485	63815917	18	AR	BBS // MKKS
WDR19	4	39183974	39287480	37	AR	Senior-Løocken // Jeune
WDR34	9	131395890	131419179	9	AR	Jeune
WHRN	9	117164310	117267786	14	AR	Usher
ZNF423	16	49524465	49891880	9	AR	Joubert

Abreviations: AD: autosomal dominant; AR: autosomal recessive; XL: X-linked; BBS : Bardet-Biedl syndrome; CRD: cone-rod dystrophy; HARP: hypoprebetalipoproteinemia, acanthocytosis, retinitis pigmentosa, palladial degeneration; LCA: Leber congenital amaurosis; MKS: Meckel syndrome; MKKS: McKusick-Kaufman syndrome; MORM: Mental retardation, truncal obesity, retinal dystrophy and micropenis; NPHP: nephronophthisis, PHARC: polyneuropathy, hypoacusia, ataxia, retinitis pigmentosa, and cataracts; ID : intellectual disability; RD: retinal dystrophy; RP: retinitis pigmentosa, S.: Syndrome, ZSSD: Zellweger syndrome spectrum disorders. Chromosome position are based on human genome build hg19.

Supplementary Table S1. Genes included in the present study (Continued).

Gene	Chromosome	Start position	End Position	No. Exons	Inheritance	Disease
Ciliary genes associated with non-syndromic RD and non-retinal ciliopathy						
ANKS6	9	101494241	101558844	15	AR	NPHP
ARL2BP	16	57278988	57287595	6	AR	Non-syndromic RP
ARL3	10	104433434	104474240	6	AR	Non-syndromic RP
C2orf71	2	29284506	29297177	2	AR	Non-syndromic RP
FAM161A	2	62051933	62081328	8	AR	Non-syndromic RP
GLIS2	16	4382175	4389648	6	AR	NPHP
KIZ	20	21106574	21227308	13	AR	Non-syndromic RCD
LCA5	6	80194658	80247197	10	AR	Non-syndromic LCA
NEK2	1	211831549	211849022	10	AR	Non-syndromic RP
NEK8	17	27055782	27069834	15	AR	Renal-hepatic-pancreatic dysplasia // NPHP
RP1	8	55528577	55543444	4	AD, AR	Non-syndromic RP
RP1L1	8	10463810	10512667	4	AD, AR	Non-syndromic MD // RP
RP2	X	46696297	46741841	5	XL	Non-syndromic RP
RPGRIP1	14	21756086	21819510	24	AR	Non-syndromic LCA
SLC41A1	1	205758171	205782211	11	AR	NPHP-like
TOPORS	9	32540492	32552676	3	AD	Non-syndromic RP
TULP1	6	35465601	35480697	15	AR	Non-syndromic RP // LCA
XPNPEP3	22	41253035	41328873	12	AR	NPHP-like

Gene	Chromosome	Start position	End Position	No. Exons	Inheritance	Disease
Syndromic vitreoretinopathies						
ADAMTS18	16	77315975	77469061	23	AR	Knobloch S., RD and autism
COL11A1	1	103341973	103574102	68	AD	Stickler
COL2A1	12	48366698	48398335	54	AD	Stickler
COL9A1	6	70925693	71012836	39	AR	Stickler
COL9A2	1	40766113	40782989	32	AR	Stickler
KCNJ13	2	233630462	233641325	5	AD, AR	Dominant vitreoretinal degeneration with systemic diseases // LCA
VCAN	5	82767443	82878172	15	AD	Wagner

Gene	Chromosome	Start position	End Position	No. Exons	Inheritance	Disease
Lysosomal and peroxisomal disorders						
CLN3	16	28488550	28503673	17	AR	Batten disease
PEX1	7	92116287	92157895	24	AR	ZSSD
PEX2	8	77892444	77913330	6	AR	ZSSD
PEX7	6	137143652	137235122	10	AR	ZSSD
PHYH	10	13319746	13342180	10	AR	Refsum disease

Abreviations: AD: autosomal dominant; AR: autosomal recessive; XL: X-linked; BBS : Bardet-Biedl syndrome; CRD: cone-rod dystrophy; HARP: hypoprebetalipoproteinemia, acanthocytosis, retinitis pigmentosa, palladial degeneration; LCA: Leber congenital amaurosis; MKS: Meckel syndrome; MKKS: McKusick-Kaufman syndrome; MORM: Mental retardation, truncal obesity, retinal dystrophy and micropenis; NPHP: nephronophthisis, PHARC: polyneuropathy, hypoacusia, ataxia, retinitis pigmentosa, and cataracts; ID : intellectual disability; RD: retinal dystrophy; RP: retinitis pigmentosa, S.: Syndrome, ZSSD: Zellweger syndrome spectrum disorders. Chromosome position are based human genome build hg19.

Supplementary Table S1. Genes included in the present study (Continued).

Gene	Chromosome	Start position	End Position	No. Exons	Inheritance	Disease
Miscellanea of syndromic retinal alteration						
ABCC6	16	16243372	16317378	32	AD, AR	Pseudoxanthoma elasticum
ABHD12	20	25275329	25371668	14	AR	PHARC S.
ACBD5	10	27484093	27531118	16	AR	Syndromic CRD
ATXN7	3	63850183	63989186	15	AD	Spinocerebellar ataxia with RD
CNNM4	2	97426589	97477678	7	AR	Jalili S.
FLVCR1	1	213031547	213072755	10	AR	RP with posterior column ataxia
GNPTG	16	1401850	1413402	11	AR	RP with spondyloepiphyseal dysplasia
JAG1	20	10618282	10654744	26	AD	Alagille S.
KIF11	10	94352775	94415202	22	AD	Chorioretinopathy with microcephaly and lymphedema
MTTP	4	100485190	100545204	19	AR	Abetalipoproteinemia and pigmentary RD
OTX2	14	57267375	57277244	8	AD	LCA with pituitary dysfunction
PANK2	20	3869436	3904552	9	AR	HARP S. // Hallervorden-Spatz
PCYT1A	3	195965203	196014634	10	AR	CRD with skeletal disease
PGK1	X	77359616	77382374	11	XL	RP with myopathy
PRPS1	X	106871604	106894306	7	XL	Neuropathy, optic atrophy, deafness, and RP
SLC9A6	X	135067533	135129478	19	XL	Christianson S. with RP
TREX1	3	48506869	48509094	5	AD	Retinal vasculopathy with cerebral leukodystrophy
TTPA	8	63971998	63998662	5	AD, AR	Spinocerebellar ataxia with RP
VPS13B	8	100025444	100889864	66	AR	Cohen S.
WFS1	4	6271527	6305042	9	AR	Wolfram S.

Abreviations: AD: autosomal dominant; AR: autosomal recessive; XL: X-linked; BBS : Bardet-Biedl syndrome; CRD: cone-rod dystrophy; HARP: hypoprebetalipoproteinemia, acanthocytosis, retinitis pigmentosa, palladial degeneration; LCA: Leber congenital amaurosis; MKS: Meckel syndrome; MKKS: McKusick-Kaufman syndrome; MORM: Mental retardation, truncal obesity, retinal dystrophy and micropenis; NPHP: nephronophthisis, PHARC: polyneuropathy, hypoacusia, ataxia, retinitis pigmentosa, and cataracts; ID : intellectual disability; RD: retinal dystrophy; RP: retinitis pigmentosa, S.: Syndrome, ZSSD: Zellweger syndrome spectrum disorders. Chromosome position are based on human genome build hg19.

Supplementary Table S2. Overview of likely pathogenic variants in autosomal recessive genes without a second likely pathogenic allele.

Gene	***cDNA***	***Protein***	***Type***	***Zygosity***	***HGMD***	***Classification***	***dbSNP***	***gnomAD***	***Phenotype of carrier individual***	***Observation***
ALMS1	c.2666C>G	p.(Ser889*)	Nonsense	HET	N	Pathogenic	NA	NA	Atypical Alström	
BBS12	c.1316_1318del	p.(Ser440del)	Inframe	HET	N	Likely pathogenic	NA	NA	RP + ID	
IFT172	c.4311+1G>T		Splicing	HET	N	Likely pathogenic	NA	NA	RP + ataxia	
MYO7A	c.1403A>G	p.(His468Arg)	Missense	HET	Y	Likely pathogenic vs VUS	rs200304238	0.00015	RP + ID + deafness	
RPGRIP1	exon 17-19 deletion		CNV	HET	N	Pathogenic	NA	2 individuals	RP + ID + various disorders	No causal. Homozygous LOF pathogenic variant found in a novel disease gene *SCAPER*[1]
PDZD7	c.642C>A	p.(Tyr214*)	Nonsense	HET	N	Pathogenic	NA	NA	RP + ID	Likely no causal variant. No phenotypic concordance with Usher syndrome
USH2A	c.5666A>G	p.(Asp1889Gly)	Missense	HET	Y	Likely pathogenic *vs* VUS	rs775803174	NA	Atypical Usher	
USH2A	c.2276G>T	p.(Cys759Phe)	Missense	HET	Y	Pathogenic	rs80338902	0.00094	Stickler (AD)	Likely no causal. No phenotypic nor inheritance concordance with Usher syndrome
WDPCP	c.208+1G>A		Splicing	HET	N	Likely pathogenic	rs187135801	0.00023	Atypical Alström	

AD: autosomal dominant inheritance; HET: heterozygous; ID: intellectual disability; LOF: loss-of- function; NA: non reported; RP: retinitis pigmentosa; VUS: variant of unknown significance.

Supplementary Table S3. Novel likely pathogenic variants found in this study.

Gene	*cDNA*	*Protein*	*Type*	*Zygosity*	Align GVGD	SIFT	MutTaster	Polyphen	CADD	Segregation	Freq. ExAC	Other predictors	Pathogenic
ABCC6	NM_001171.5:c.1483_1485del	p.(Leu495del)	In-frame deletion	HOM	----	----	----	----	37	NA	NA	D (PROVEAN)	Likely
ALMS1	NM_015120.4:c.4252del	p.(Arg1418Glyfs*55)	Frameshift	HOM	----	----	----	----	38	Y	NA		Y
ALMS1	NM_015120.4:c.2666C>G	p.(Ser889*)	Nonsense	HET	----			----	38	Y	NA		Y
BBS12	c.1316_1318del	p.(Ser440del)	In-frame deletion	HET	----			----	12.75	NA	NA	D (PROVEAN)	Likely
CEP41	NM_018718.2:c.5C>T	p.(Ser2Phe)	Missense	HOM	C25	D	D	Probably damaging	26.3	NA	0.028%		Likely
IFT172	c.4311+1G>T	Splicing defect	Splice donor	HET	----	----	----	----	25.2	NA	NA	100% variation for donor site	Likely
IFT27	NM_006860.4:c.104A>G	p.(Tyr35Cys)	Missense	HET *	C65	D	D	Probably damaging	29.2	NA	NA		Likely
IFT27	NM_006860.4:c.350-2A>G	Splicing defect	Splice acceptor	HET *	----		----	----	23.3	NA	NA	100% variation for acceptor site	Y
OTX2	c.559C>T	p.(Gln187*)	Nonsense	HET	----	----	----	----	23.2	*de novo*	Y		Y
OTX2	c.255G>A	p.(Trp85*)	Nonsense	HET	----	----	----	----	24.4	*de novo*	NA		Y
PDZD7	c.642C>A	p.(Tyr214*)	Nonsense	HET	----	----	----	----	5385	NA	NA		Y
VPS13B	c.1512del	p.(Glu505Lysfs*23)	Frameshift	HOM	----	----	----	----	29.8	Y	NA		Y
WDPCP	c.208+1G>A	Splicing defect	Splice donor	HET	----	----	----	----	33	NA	0.00023	100% variation for donor site	Likely

The novel variants were not found in several databases, including HGMD, ExAC and the Spanish Variant Server. Aminoacid substitution in missense variant is predicted damaging by SIFT if the score is <= 0.05 and PROVEAN score is < -2.5; Polyphen predicts a non-synonymous variant as benign, possibly damaging, or probably damaging, if score is < 0.2, between 0.2 and 0.85 or > 0.85; GV: Grantham Variation; GD: Grantham distribution. Class C65: most likely pathogenic, Class C0: less likely pathogenic; Mutation Taster: deleterious > 0.65; CADD: deleterious > 15. *: Variant found in *trans* with a second pathogenic allele. Abbreviations: HOM: homozygous; HET: heterozygous; NA: not available; Y: considered as pathogenic.

Supplementary Table S4. Deep intronic variants included in the gene panel.

Gene	Genomic coordinates (hg19)	cDNA	Reference
CEP290	chr12:88494960	NM_025114: c.2991_1655A>G; p.(Cys998*)	2
USH2A	chr1:216064540	NM 206933.2: c.7595-2144A>G	3
OFD1	chrX:13768358	NM_003611.2: c.935+706A>G	4
BBS1	chr11:66291105	NM_024649.4: c.951+58C>T	5

REFERENCES

1 Tatour, Y. et al., Mutations in SCAPER cause autosomal recessive retinitis pigmentosa with intellectual disability. *J Med Genet* (2017).

2 den Hollander, A. I. et al., Mutations in the CEP290 (NPHP6) gene are a frequent cause of Leber congenital amaurosis. *Am J Hum Genet* **79** (3), 556 (2006).

3 Vache, C. et al., Usher syndrome type 2 caused by activation of an USH2A pseudoexon: implications for diagnosis and therapy. *Hum Mutat* **33** (1), 104 (2012).

4 Webb, T. R. et al., Deep intronic mutation in OFD1, identified by targeted genomic next-generation sequencing, causes a severe form of X-linked retinitis pigmentosa (RP23). *Hum Mol Genet* **21** (16), 3647 (2012).

5 Abu Safieh, L. et al., Clinical and molecular characterisation of Bardet-Biedl syndrome in consanguineous populations: the power of homozygosity mapping. *J Med Genet* **47** (4), 236 (2009).

Supplementary Table S5. Previously known variants used as controls.

Gene	RefSeq	cDNA	Protein	Method	refSNP	Effect
BBS2	NM_031885.3	c.209A>G	p.(Asn70Ser)	BBS Chip, Sanger	rs4784677	Likely pathogenic
ALMS1	NM_015120.4	c.5465C>T	p.(Pro1822Leu)	BBS Chip, Sanger	rs238997	VUS
CEP290	NM_025114.3	c.4028delA	p.(Lys1343Argfs*2)	LCA Chip, Sanger	rs1057519245	Likely pathogenic
BBS1	NM_024649.4	c.1645G>T	p.(Glu549*)	BBS Chip, Sanger	rs121917777	Pathogenic
MKKS	NM_170784.2	c.724G>T	p.(Ala242Ser)	BBS Chip, Sanger	rs74315394	Likely benign
CDH23	NM_001171930.1	c.1096G>A	p.(Ala366Thr)	Usher Chip, Sanger	rs143282422	Likely benign

Abbreviations: BBS: Bardet-Biedl syndrome; LCA: Leber congenital amaurosis; VUS: variant of unknown significance.
